# Immersive learning in obstetrics: a 360-degree video for teaching the management of premature rupture of membranes

**DOI:** 10.1590/1806-9282.20251605

**Published:** 2026-06-19

**Authors:** Eduardo Moreira Nader, Ana Beatriz Ramos Antiqueira, Roseli Mieko Yamamoto Nomura

**Affiliations:** 1 Universidade Federal de São Paulo, Escola Paulista de Medicina, Department of Obstetrics, Division of Experimental and Physiologic Obstetrics – São Paulo (SP), Brazil.

**Keywords:** Education, medical, Simulation training, Virtual reality, Obstetrics, Fetal membranes, premature rupture

## Abstract

**OBJECTIVE:**

The aim of this study was to evaluate the performance and perception of a 360-degree video as an educational tool for medical students in the management of premature rupture of membranes.

**METHODS:**

A prospective interventional study was conducted with fifth-year medical students. Participants watched a 10-min, 360-degree video simulating a complete premature rupture of membranes case, from anamnesis to prescription. Student perceptions were assessed using a questionnaire with a five-point Likert scale, covering technical quality, comprehension of clinical procedures, and pedagogical impact. A subgroup analysis compared outcomes based on the viewing device (mobile phone vs. computer).

**RESULTS:**

A total of 60 medical students were included in this study. The video intervention received a highly favorable evaluation. Students reported high rates of comprehension for key clinical decision-making elements, including the indication for hospital admission (60 students, 100%) and the rationale for antibiotic therapy (59 students, 98.3%). The experience positively influenced student motivation (53 students, 88.3%) and heightened their interest in obstetrics (45 students, 75.0%). No statistically significant (p>0.05) differences were found between the proportion of positive responses (agree and strongly agree) according to the device used to watch the 360-degree video: mobile phone (n=34, 56.7%) vs. computer (n=26, 43.3%).

**CONCLUSION:**

The use of 360-degree video is an effective and well-received pedagogical tool for teaching complex obstetric scenarios. Its comparable effectiveness on both mobile and computer platforms underscores its potential as an accessible and scalable resource to better prepare students for the practical challenges of the obstetrics and gynecology clerkship.

## INTRODUCTION

Since the beginning of the new millennium, educators around the world have been exploring new teaching methods, recognizing that students born in this century—commonly referred to as digital natives—require more interactive and diverse pedagogical approaches^1^. This challenge is particularly pronounced in medical education, where the ability to capture and maintain students’ attention has become crucial to an effective teaching–learning process^2^.

In this context, the use of innovative technologies, such as 360-degree video, has emerged as a promising tool for improving students’ visual and auditory immersion in the learning environment^3^. By allowing simultaneous viewing of all perspectives, 360-degree video transcends the limitations of traditional two-dimensional videos, aiming for greater interactivity and viewer engagement^4^.

The importance of new technologies in medical education has become even more relevant after the COVID-19 pandemic, which accelerated the transition from face-to-face teaching models to distance learning models, highlighting the fundamental role of digital media in medical training^5^. In the specific field of obstetrics, where students’ exposure to practical situations can have implications for maternal and fetal health, virtual simulation has been recognized as a beneficial and safe approach^6,7^.

The visual and auditory immersion provided by 360-degree video presents itself as an alternative for teaching, capable of overcoming distractions, and as a valuable tool for engaging students^8^. Recent studies indicate that the use of 360-degree videos in health education promotes improvements in student motivation and self-confidence. These improvements are attributed to a greater sense of “presence” and immersion in the learning environment, which are fundamental to the consolidation of skills^9,10^.

Although there is growing adoption of this technology for emergency training^10^, there is a gap in teaching the management of obstetric conditions such as premature rupture of membranes (PROM). Therefore, this study focuses on the use of 360-degree video as a teaching resource, exploring its performance as a facilitator of obstetric care learning. The objective is to evaluate the performance and perception of a 360-degree video as an educational tool for medical students in the treatment of PROM.

## METHODS

This is a prospective interventional study addressing the simulation of management of PROM. The research protocol was approved by the Institutional Research Ethics Committee (CAAE Number 57376822.0.0000.5505), and all the participants signed a consent form.

A 10-min 360-degree video was produced using a Samsung Gear 360 camera. The video was developed at the Simulation Center at the Federal University of São Paulo, in a facility equipped for virtual media creation. In the simulation session, a NoelleS575, a full-sized female anthropomorphic birth simulator, and a model of cervical dilation (Life/form^®^ Cervical Dilatation and Effacement Simulator) were used. The room in the simulation lab is specially prepared to manage obstetric cases. For the scenarios that simulate a woman’s admission, standardized speeches were prepared with specific topics challenging the student caring for the simulated patient complaining of rupture of membranes. This scenario was developed with the participation of an actress (a graduate student) representing a woman at 39 weeks’ gestation who tests positive for Group B streptococcus. She complains of fluid loss from her vagina that started 8 h before and is now experiencing contractions every 30 min. The doctor conducts an anamnesis to establish the full extent of the situation, including the woman’s expectations. A physical examination was performed on the fullsized female simulator to verify maternal and fetal status and to reach a clinical diagnosis by conducting a speculum examination and using diagnostic tests. Furthermore, the Bishop’s index was calculated to determine obstetric management and perform a medical prescription. In this scenario, a 2 cm cervical dilation effacement simulator and a medical prescription form were used. In addition, the interpretation of the cardiotocography performed on the patient was demonstrated.

Between August 2022 and November 2023, fifth-year undergraduate medical students were invited to participate during the 4-week clerkship rotation in their 5th year. The 360-degree video was made available on private YouTube, and access was allowed through the link provided in an electronic form sent via WhatsApp. The questionnaire consists of the following items: age, skin color, device used to watch the video (cell phone, personal computer, or tablet), and opinion on the length of the video. The items in the questionnaire were scored using a five-point Likert scale (strongly disagree, disagree, neutral, agree, strongly agree). Regarding the technical aspects of the 360° video, students were asked about the quality of the images for adequate viewing, the quality of the audio for understanding the dialogues, the possibilities for navigating the virtual environment, and the suitability of the video’s availability. Concerning the perception of procedures, eight questions addressed the obstetric physical examination and eight questions focused on clinical management. Finally, four questions were included about the availability of the video and the students’ interest in the topics covered.

Data were analyzed using the MedCalc^®^ Statistical Software version 19.5.3 (MedCalc Software Ltd., Ostend, Belgium; 2020). Descriptive statistics are presented as mean and standard deviation (SD), frequency, and percentage (%). To assess the internal consistency of the instrument used, Cronbach’s alpha was calculated, yielding a value of 0.831, indicating high reliability (95% lower confidence limit: 0.774). The comparison between proportions was drawn using the chi-square test or Fisher’s exact test. Statistical significance was set at p<0.05.

## RESULTS

A total of 130 students were invited, and 60 agreed to participate (46.2%). The demographic characteristics of the participants and how they accessed the video are detailed in Table 1.

**Table 1:** Characteristics of medical students who evaluated the 360-degree video on the management of premature rupture of membranes (n=60).

Characteristics	Results
Age (years), mean (SD)	25.7 (4.1)
Gender, n (%)	
Female	27 (45)
Male	33 (55)
Race/ethnicity, n (%)	
White	38 (63.3)
Mixed	11 (18.3)
Asian	9 (15)
Black	2 (3.3)
Device, n (%)	
Mobile phone	34 (56.7)
Personal computer	26 (43.3)

SD: standard deviation.

The students’ perceptions of the technical attributes of the video, their evaluation of the procedures demonstrated, and their interest in the topic are presented in Table 2. The technical evaluation of the 360-degree video was highly favorable. Most students rated the image and audio quality as satisfactory or superior. Similarly, access and navigation in the virtual environment were evaluated positively. Most students understood the steps involved in the abdominal physical examination. In contrast, procedures involving the pelvic examination generated more varied responses in terms of clarity. Clinical decision-making was also well understood, and a high proportion of participants correctly identified the indication for hospitalization, the absence of an indication for immediate cesarean section, and the justification for antibiotic therapy. Self-reported data indicated an increase in involvement in learning about PROM and a general interest in obstetrics as a specialty.

**Table 2:** Medical students’ assessment of the technical aspects, procedures, clinical reasoning, and pedagogical impact of the 360-degree video on the management of premature rupture of membranes (n=60).

Video topics evaluated	Disagree/neutral	Agree
Technical aspects		
Appropriate image quality	8 (13.3)	52 (86.7)
Clear audio for dialogue comprehension	0 (0)	60 (100)
Navigation capabilities met expectations	13 (21.7)	47 (78.3)
Appropriateness of video delivery method	2 (3.3)	58 (96.7)
Clinical procedures and communication		
Noticeable physician-to-patient care	0 (0)	60 (100)
Clarity of fundal height measurement	4 (6.7)	56 (93.3)
Clarity of the four Leopold’s maneuvers	2 (3.3)	58 (96.7)
Comprehension of FHR auscultation (Pinard)	4 (6.7)	56 (93.3)
Clarity of uterine dynamics assessment	4 (6.7)	56 (93.3)
Comprehension of the pelvic exam	7 (11.7)	53 (88.3)
Comprehension of the nitrazine test	9 (15.0)	51 (85.0)
Comprehension of the phenol red test	7 (11.7)	53 (88.3)
Clinical reasoning and management		
Noticed that cesarean section was not indicated	0	60 (100)
Comprehension of the need for admission due to ruptured membranes	0	60 (100)
Comprehension of the reason for antibiotic prescription	1 (1.7)	59 (98.3)
Noticed that fetal well-being was explained to the patient	2 (3.3)	58 (96.7)
Understanding the prescription at admission	7 (11.7)	53 (88.3)
Pedagogical impact		
Belief that the video will aid learning	1 (1.7)	59 (98.3)
Increased interest in learning about PROM	7 (11.7)	53 (88.3)
Increased interest in obstetrics	15 (25.0)	45 (75.0)

Data presented as n (%). PROM: premature rupture of membranes; FHR: fetal heart rate.

Table 3 compares the proportion of positive responses (agree and strongly agree) according to the device used to watch the 360-degree video (mobile phone vs. computer). No statistically significant differences were found between these groups, suggesting that the immersive experience and levels of understanding and engagement were comparable regardless of the device used.

**Table 3:** Characteristics of medical students and comparison of the proportion of positive responses according to the device used to watch the 360-degree video on the management of premature rupture of membranes.

Parameter	Mobile phone (n=34)	Computer (n=26)	p-value
Students’ characteristics			
Age, mean (SD)	25.9 (4.1)	25.4 (4.1)	0.446
Race, white, n (%)	23 (67.6)	15 (57.7)	0.841
Technical aspects			
Appropriate image quality	30 (88.2)	22 (84.6)	0.685
Clear audio for dialogue comprehension	34 (100)	26 (100)	0.302
Navigation capabilities met expectations	27 (79.4)	20 (76.9)	0.818
Appropriateness of video delivery method	33 (97.1)	25 (96.2)	0.848
Clinical procedures & communication			
Noticeable physician-to-patient care	34 (100)	26 (100)	0.302
Clarity of fundal height measurement	32 (94.1)	24 (92.3)	0.782
Clarity of the four Leopold’s maneuvers	33 (97.1)	25 (96.2)	0.848
Comprehension of FHR auscultation (Pinard)	31 (91.2)	25 (96.2)	0.448
Clarity of uterine dynamics assessment	33 (97.1)	23 (88.5)	0.190
Comprehension of the pelvic exam	30 (88.2)	23 (88.5)	0.979
Comprehension of the nitrazine test	30 (88.2)	21 (80.8)	0.426
Comprehension of the phenol red test	30 (88.2)	23 (88.5)	0.979
Clinical reasoning & management			
Noticed that cesarean section was not indicated	34 (100)	26 (100)	0.302
Comprehension of the need for admission due to ruptured membranes	34 (100)	26 (100)	0.302
Comprehension ofthe reason for antibiotic prescription	33 (97.1)	26 (100)	0.382
Noticed that fetal well-being was explained to the patient	34 (100)	24 (92.3)	0.103
Understandingthe prescription at admission	32 (94.1)	21 (80.8)	0.114
Pedagogical impact			
Belief that the video will aid learning	34 (100)	25 (96.2)	0.253
Increased interest in learning about PROM	31 (91.2)	22 (84.6)	0.437
Increased interest in obstetrics	27 (79.4)	18 (69.2)	0.371

Data presented as n (%). SD: standard deviation; PROM: premature rupture of membranes; FHR: fetal heart rate.

## DISCUSSION

The 360-degree video proved to be a teaching tool in PROM management for medical students. The video intervention received a highly favorable evaluation, and students reported high rates of comprehension for key clinical decision-making elements. The results reveal that the tool was able to transmit essential information, allowing students to achieve a comprehensive understanding. These results suggest that this immersive technology plays a significant role as a pedagogical complement.

The results of this research support growing evidence of the benefits of immersive technologies in medical education. A study evaluating the use of 360-degree video for learning laparoscopic cholecystectomy demonstrated that this method generated a significantly higher degree of engagement and attention when compared to traditional two-dimensional video^3^. Notably, the authors observed that students’ attention was not only higher but also increased progressively throughout the viewing, an effect rarely seen in conventional teaching formats, such as didactic lectures^11^. This potential to maintain and increase engagement, combined with positive feedback from participants, who described it as beneficial and even “fun,”^12^ highlights the intrinsic value of the 360-degree format for complex learning.

In obstetrics, the literature highlights the impact of technology on professional training. In an experimental study with healthcare professionals, Yang et al.^13^ used a virtual reality video to train participants in the management of breech deliveries and observed a significant increase in participants’ self-confidence after the intervention. All participants rated the technology as a valuable tool for consolidating their learning.

The literature widely recognizes that the experience during the obstetrics and gynecology internship is one of the most decisive factors in the student’s perception of their eventual choice of specialty^14^. Although many external factors influence this decision, a positive internship experience can influence students’ interest, while negative experiences or learning barriers can be a strong impediment^15^.

A Canadian study of medical students reported that, during a 6-week internship, students were refused when attempting to perform a pelvic exam. This difficulty is significantly more pronounced for male students, who report being refused more often than their female colleagues, both by patients and by preceptors who are hesitant to introduce them^16^. Danielsson et al.^17^ confirmed that while female students perceive their gender as an advantage, 98% of male students who noticed a gender influence considered it negative.

Teaching tools such as 360-degree videos demonstrate strategic value in reducing initial barriers to learning. By offering virtual immersion in a realistic and standardized clinical setting, the approach allows all students to experience the complete care offered to a patient with PROM. This experience can build a cognitive and confidence base, better preparing students to optimize the practical opportunities they will eventually receive, potentially improving their performance and the safety conveyed to the mother– fetus dyad^18^.

This study has limitations. Not all students have the same equipment to access 360-degree video, which may vary according to technical capacity in data transmission, which can compromise immersion in the scenario. The actual capacity for learning and long-term knowledge retention was also not investigated, aspects that should be studied in the future. In addition, no specific cognitive assessment of learning about the topic covered was performed.

In conclusion, the use of 360-degree video is an important and well-received pedagogical tool for complex obstetric scenarios. The integration of 360-degree video into the curriculum for teaching the clinical management of PROM is a beneficial strategy. Its performance is comparable on mobile and computer platforms, pointing to its potential as an accessible resource for preparing students for the practical challenges of their obstetrics and gynecology internship.

This study was supported by a scientific initiation scholarship from the São Paulo Research Foundation (FAPESP, process no. 2022/01592-8).

## Data Availability

The datasets generated and/or analyzed during the current study are available from the corresponding author upon reasonable request.
